# A personalized, web-based breast cancer decision making application: a pre-post survey

**DOI:** 10.1186/s12911-019-0924-7

**Published:** 2019-10-21

**Authors:** Kirk D. Wyatt, Sarah M. Jenkins, Matthew F. Plevak, Marcia R. Venegas Pont, Sandhya Pruthi

**Affiliations:** Mayo Clinic, 200 First Street SW, Rochester, MN 55905 USA

**Keywords:** Breast cancer, Mobile applications, Clinical decision-making, Shared decision making

## Abstract

**Background:**

Every case of breast cancer is unique, and treatment must be personalized to incorporate a woman’s values and preferences. We developed an individually-tailored mobile patient education application for women with breast cancer.

**Methods:**

Pre-post surveys were completed by 255 women who used the tool.

**Results:**

Patients thought the application included helpful information (*N* = 184, 72%) and was easy to navigate (*N* = 156, 61%). Most patients thought the amount of information in the tool was “about right” (*N* = 193, 87%). Decision making confidence increased by an average of 0.8 points (10-point scale) following a consultation and use of the tool (*p* < 0.001).

**Conclusions:**

Tailored mobile applications may optimize care by facilitating shared decision making and knowledge transfer, and they may also enhance the experience of patients as they navigate through their breast cancer journey.

## Background

Breast cancer is one of the most common forms of cancer [1], and patients may be overwhelmed by the information they are provided at the time of diagnosis. Individually-tailored, electronically-delivered education and decision making support may facilitate decision making and increase patient satisfaction. In this section, we will discuss breast cancer treatment decision making, tailored patient education and electronic patient education delivery methods.

### Breast cancer

Breast cancer is the most common cause of cancer in women [1]. The most recent report from the American Cancer Society [1] estimates that over a quarter-million women are diagnosed with breast cancer in the United States annually, and over 40,000 women will die this year due to breast cancer.

Treatment of breast cancer is personalized and requires incorporation of a woman’s values and preferences as well as individual characteristics about her specific cancer diagnosis. Patients generally undergo surgery and may undergo other therapies including chemotherapy, hormonal therapy and radiotherapy [2]. Surgical decisions for early stage breast cancer tend to be heavily based on each patient’s personal values and preferences. These include the decision of mastectomy vs. lumpectomy, whether to pursue contralateral prophylactic mastectomy, and whether to pursue reconstructive surgery. These decisions require women to weigh the risks and benefits, recovery time and short- and long-term effects associated with the different surgical options. Furthermore, the use of adjuvant therapies may depend on the biology of a woman’s breast cancer, such as hormone receptor or HER2 status and staging [2].

### Patient education and decision making

With so many choices to be made, the treatment decision making process can be overwhelming. Traditionally, patient education and decision making has been a process involving face-to-face discussions with a nurse and a physician. Barriers to educating patients with breast cancer include emotional distress and lack of access to accurate and comprehensive information [3]. Because patients may only retain a limited amount of the information discussed during a clinical consultation, patients are often provided with post-visit patient education materials, generally in printed form. One significant limitation of this approach is that patient education materials are usually not tailored to an individual patient’s context and may describe extraneous treatment options that do not apply to the patient, thereby compounding the problem of information overload and causing confusion.

Shared decision making has emerged as the preferred approach for complex decisions for which there is not one single-best treatment option and where the optimal treatment option for each patient may depend on a number of characteristics. Shared decision making is often facilitated using decision aids. A recent systematic review highlighted 23 decision aids for women facing treatment decisions about breast cancer [4]. Decision aids for women with breast cancer appeared to decrease decisional conflict and increase knowledge and satisfaction, though anxiety did not appear to be affected [4].

### Internet-based patient education

Over 70% of patients with breast cancer seek information about their diagnosis online [5]; however, the quality of information provided in online resources can be highly variable, and these resources may be unreliable [5–7]. Because web-based education may make patients more comfortable making treatment decisions and may decrease anxiety [8], our clinic previously explored the use of an algorithmic web-based patient education tool used during consultations in our breast cancer clinic and demonstrated favorable results [9]. A similar approach has been reported in Newfoundland and Labrador [10].

### Study introduction

We identified a need to improve patient education and decision making confidence by providing reputable information in a manner that is personalized to the patient’s individual tumor type and biology. Consistent with the interdisciplinary treatment approach at Mayo Clinic, we developed a comprehensive, interactive, personalized educational tool delivered on a mobile device to assist our Breast Clinic patients with treatment decision making and to empower them to engage in shared decision making. In order to assess the feasibility and utility of this approach, we conducted patient surveys before and after use of the tool.

## Methods

The study involved development of a web-based application designed for use on a tablet computer. Patients with newly-diagnosed breast cancer were provided with tablet computers and encouraged to use the application. The impact on patient-centered outcomes was assessed using surveys conducted before and after use of the application. Herein, we describe these procedures in further detail.

### Study description

The aim of this study was to gather initial data to assess the utility, ease of use and impact of a mobile web-based tool on decision making confidence. The study was conducted within the interdisciplinary Breast Clinic at Mayo Clinic, which serves a large referral population. The tool was integrated into the Breast Clinic as standard-of-care. Pre-post surveys were administered to all patients at the Breast Clinic who used the application and agreed to take part in the surveys before using the application and following use of the application.

### Application development

After conducting patient focus groups to identify functional and non-functional requirements, a mobile web application was developed with the assistance of a third-party company (TakeTheWind, Coimbra, Portugal). Content was curated by an interdisciplinary team of breast cancer experts. Materials were written at a 6th to 8th grade reading level with the assistance of Mayo Clinic Patient Education. The application was designed for use on an iPad (Apple, Inc., Cupertino, California) tablet utilizing the native Safari browser in landscape orientation and requires an active internet connection. The application was coded utilizing the Yii framework, including the languages PHP and Javascript. Data were stored in a MySQL database. We chose a web-based application to ensure data security and to facilitate real-time content updates. The application was not designed for use in other web browsers or on other devices, and it was separate from the institutional patient online medical record portal. The application received approval through our formal institutional security and data privacy review process. The user portal was password-protected. For data security purposes, limited patient identifiers were collected, and one-way cryptographic hash functions were used to maintain data security.

### Application implementation

Ninety tablets were purchased and loaded with the application. Participants were offered a tablet during their initial clinical visit in the Breast Diagnostic Clinic at Mayo Clinic in Rochester, Minnesota. Patients were allowed to keep the tablet until after their surgical treatment, which was usually completed between one and four weeks after their initial visit. Tablet computers were chosen as the delivery method to ensure a consistent experience across all patients and to ensure that personal computer ownership and access were not barriers to use.

### Application features

The application included information regarding breast anatomy, definition of breast cancer, breast cancer types, tumor grade, tumor markers, and breast cancer staging. In addition, the tool included information about the risks, benefits, timelines and recovery for each of the medical, surgical and radiation therapy options. A physician dashboard allowed the breast specialist to enter patient-specific information (e.g., breast cancer type, hormone receptor status, grade, and stage) and pertinent therapeutic options, based on the initial consultation, which subsequently tailored the content that was visible to the patient.

**Fig. 1:**
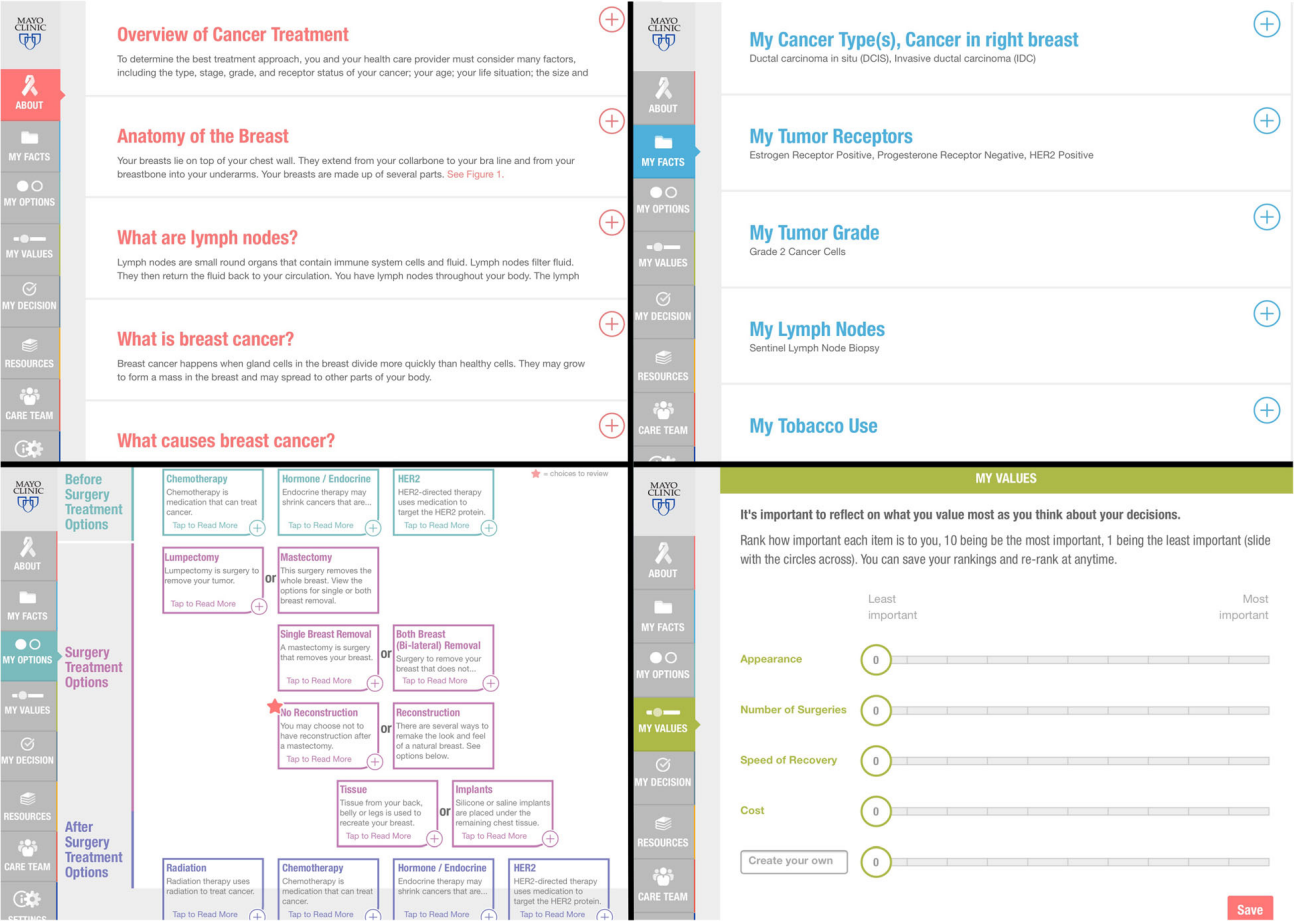
Application screenshots

When patients initially logged in, they were presented with a brief, 2-question survey assessing how they preferred to make health care decisions. The interactive tool enabled patients to state their values and their level of confidence in making a treatment decision using a Likert scale from 1 to 10, with 1 signifying not at all confident and 10 signifying extremely confident. The health care team was able to view these patient responses on the dashboard at their discretion. The tool also included photographs of the interdisciplinary breast team, including surgeons, physicians, advanced practice providers, and nursing staff. Screenshots of the educational tool are shown as Fig. 1.

### Patient surveys

**Fig. 2:**
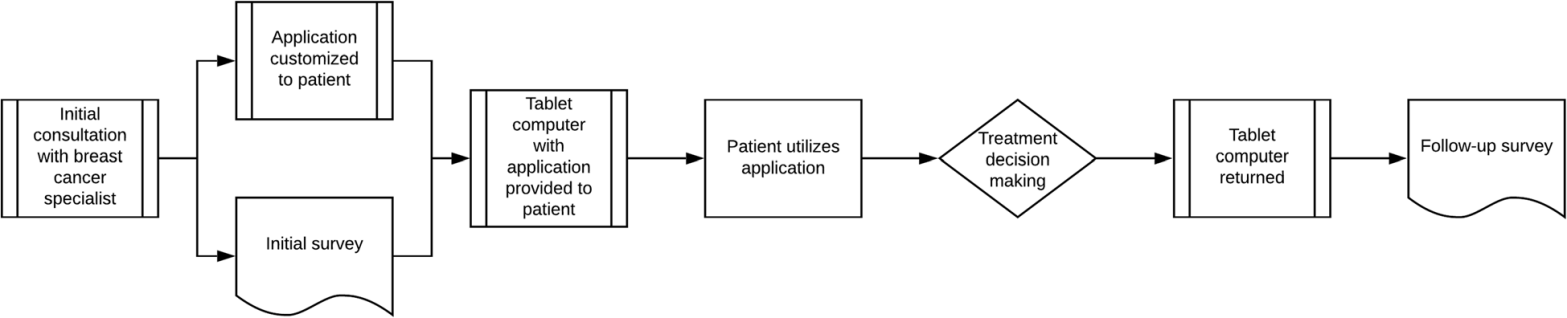
Survey workflow

Patient surveys were administered at in-person clinical visits prior to and after use of the educational tool (Fig. 2). Due to the evolution of our workflows, not all patients completed both surveys, and two slightly different versions of the post-intervention survey were administered during the period of study. Survey questions were adapted from a previous survey conducted on patients who had used a computerized breast cancer tool at our institution [9]. Pre-intervention surveys asked patients to rate their preferred role on a five-point scale ranging from independently making the decision, to shared decision making and to the doctor independently making the decision. Patient confidence was rated on a 1 to 10 scale with higher numbers signifying greater confidence.

In post-intervention surveys, users who had used the educational tool were asked to share reasons why the tool was useful, and users who had not used the tool were asked why the tool was not useful. Patients were then asked to report the extent the tool increased understanding of breast cancer, whether they would recommend the tool to other patients newly diagnosed with breast cancer, and how confident they were in their final decision regarding treatment. Patients were also asked to rate the overall amount of educational information on a three-point scale (not enough, about right, too much).

### Data extraction from the electronic health record

In order to understand whether demographic and disease-specific factors affect outcomes, we extracted relevant attributes from the medical record, including patient age at diagnosis, distance from clinic to home zip code, education level, type of breast cancer, staging information, and type of treatment(s) and/or surgery received. Most information was extracted from an integrated breast cancer treatment summary document.

### Statistical procedures

The data from the pre- and post-intervention surveys were summarized with frequencies and percentages, medians and interquartile ranges (IQR), or means and standard deviations (SD) as appropriate. Baseline demographics and clinical characteristics were compared between those who used versus did not use the educational tool (intervention) with chi-square tests or Fisher’s exact tests for categorical variables, and with Wilcoxon rank-sum tests or two-sample t-tests for ordinal or continuous variables. Confidence in the ability to make treatment decisions was compared between time points with a paired t-test. The average pre-intervention confidence level and the average change in confidence level were compared between groups with ANOVA F-tests. Multivariable linear regression was used to examine associations with change in confidence, adjusting for pre-intervention confidence, distance to the clinic, cancer type, cancer stage, preferred role in decision making, and education level. The Pearson correlation coefficient (r) was used to quantify the linear association between pre-intervention with post-intervention confidence, as well as pre-intervention with change in confidence. All analyses were performed using SAS 9.4 (SAS Institute Inc., Cary, North Carolina).

## Results

After excluding 23 patients (100% female; median age 61 years, range 33–77) who requested their medical records not be used for research purposes, we identified 447 patients who completed the post-intervention survey and 290 patients who completed both the pre-intervention and post-intervention survey. In the former group, 390 (87%) of patients reported using the application and in the latter group 255 (88%) of patients reported using the application (Table 1). Demographics and survey responses for these patients will be discussed below.

**Table 1:** Patient demographics and treatments pursued

	Used tool (*N* = 255)^a^	Did not use tool (*N* = 35)^a^	Total (*N* = 290)^a^
Age in years (mean [SD])	56.8 (11.8)	60.6 (12.0)	57.2 (11.8)
Race			
White	236 (92.5%)	31 (88.6%)	267 (92.1%)
African American	1 (0.4%)	1 (2.9%)	2 (0.7%)
American Indian/Alaskan Native	4 (1.6%)	0 (0.0%)	4 (1.4%)
Asian	7 (2.7%)	2 (5.7%)	9 (3.1%)
Other	2 (0.8%)	0 (0.0%)	2 (0.7%)
Unknown or choose not to disclose	5 (2.0%)	1 (2.9%)	6 (2.1%)
Education			
Some high school (HS), did not graduate	1 (0.4%)	0 (0.0%)	1 (0.3%)
HS graduate or GED	35 (13.8%)	12 (35.3%)	47 (16.3%)
Some college or 2-year degree	81 (31.9%)	8 (23.5%)	89 (30.9%)
4-year college graduate	69 (27.2%)	8 (23.5%)	77 (26.7%)
Post graduate studies	68 (26.8%)	6 (17.6%)	74 (25.7%)
Preferred decision making approach			
I make decision	5 (2.2%)	3 (8.6%)	8 (3.0%)
I make decision & consider doctor’s opinion	68 (29.4%)	11 (31.4%)	79 (29.7%)
Shared decision making	144 (62.3%)	18 (51.4%)	162 (60.9%)
Doctor makes decision & considers my opinion	11 (4.8%)	3 (8.6%)	14 (5.3%)
Doctor makes decision	3 (1.3%)	0 (0.0%)	3 (1.1%)
Breast cancer stage			
Stage 0	35 (16.2%)	3 (12.0%)	38 (15.8%)
Stage I	97 (44.9%)	13 (52.0%)	110 (45.6%)
Stage II	66 (30.6%)	5 (20.0%)	71 (29.5%)
Stage III	18 (8.3%)	4 (16.0%)	22 (9.1%)
Breast cancer laterality			
Bilateral	11 (4.9%)	4 (14.8%)	15 (5.9%)
Unilateral	215 (95.1%)	23 (85.2%)	238 (94.1%)
Breast cancer type			
Bilateral (discordant)	6 (2.7%)	1 (3.7%)	7 (2.8%)
Ductal carcinoma in situ	39 (17.3%)	3 (11.1%)	42 (16.7%)
Invasive	180 (80.0%)	23 (85.2%)	203 (80.6%)
Hormonal therapy^b^			
Adjuvant	139/226 (61.5%)	17/27 (63.0%)	156/253 (61.7%)
Neoadjuvant	13/226 (5.8%)	0/27 (0.0%)	13/253 (5.1%)
Chemotherapy^b^			
Adjuvant	36/226 (15.9%)	6/27 (22.2%)	42/253 (16.6%)
Neoadjuvant	13/226 (5.8%)	0/27 (0.0%)	13/253 (5.1%)
Radiation therapy^b^	133/226 (58.8%)	18/27 (66.7%)	151/253 (59.7%)
Surgery^b^			
Lumpectomy	123/226 (54.4%)	14/27 (51.9%)	137/253 (54.2%)
Unilateral mastectomy	44/226 (19.5%)	4/27 (14.8%)	48/253 (19.0%)
Bilateral mastectomy	59/226 (26.1%)	8/27 (29.6%)	67/253 (26.5%)
Reconstructive surgery	75/226 (33.2%)	6/27 (22.2%)	81/253 (32.0%)

^a^Frequencies not adding to column total indicate missing data (not included in denominator for percentages)

^b^Categories not mutually exclusive (denominators provided)

### Demographics

Patient demographics are summarized in Table 1. All of the included patients were females, 92% were white, and most (83%) had at least some college education. Ages were approximately normally distributed with a mean age of 57 years (SD 11.8 years, range 29–85).

**Fig. 3:**
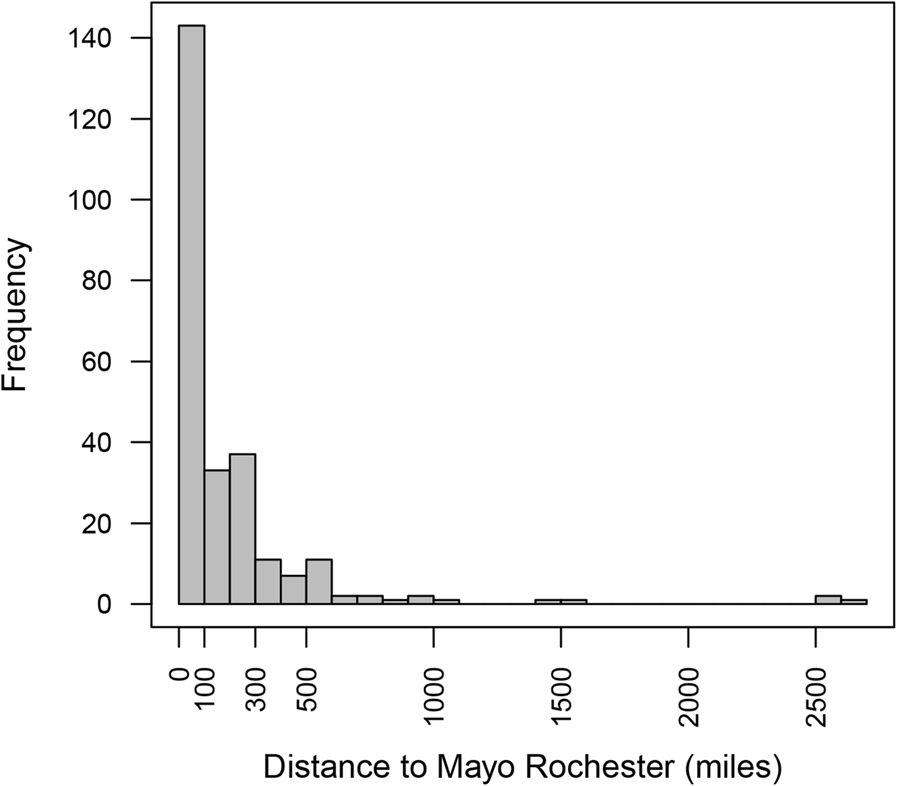
Distance between patients’ homes and our clinic

Most patients (77%) lived more than 30 miles from downtown Rochester, Minnesota, with nearly one quarter (23%) living more than 250 miles away. The median distance was 81 miles. A histogram showing distance between the patient’s home address and our clinic is demonstrated as Fig. 3.

Abstracted clinical data were available for 253 patients (cancer type, laterality, treatment, and surgery type), with cancer stage available for 241 patients (Table 1). Six percent of patients had bilateral breast cancer. The majority (80%) had invasive cancer. Seventeen percent had ductal carcinoma in situ, and 3% had bilateral cancers with ductal carcinoma in situ on one side and invasive cancer on the other side (i.e., discordant). The majority of patients were classified as either Stage 0 (16%) or Stage I (46%).

The baseline demographic and clinical characteristics of patients who were offered but did not use the educational tool were similar to those who had used the educational tool with the exception of education. Patients who used the tool had higher levels of education as compared to the non-users (86% vs 65% with at least some college, *p* = 0.03) (Table 1).

### Treatments received

Treatments received by patients are summarized in Table 1. In terms of hormonal therapy, neoadjuvant therapy was received by 5% of patients, and adjuvant therapy was received by 62%. In terms of chemotherapy, neoadjuvant therapy was received by 16% of patients, and adjuvant therapy was received by 17% of patients. Sixty percent of patients received radiation.

In terms of surgical management, 54% of women underwent lumpectomy, 19% underwent unilateral mastectomy, and 27% underwent bilateral mastectomy. Thirty two percent of patients underwent reconstructive surgery.

### Preferred approach to decision making

Most patients preferred to engage in shared decision making when making treatment decisions. The distribution of preferred decision making approaches is shown in Table 1.

### Facilitators and barriers to use

In the post-intervention survey, users who had used the educational tool were asked to share reasons why the tool was useful, and users who had not used the tool were asked why the tool was not useful. As we analyzed survey responses, it became apparent that many patients ignored the intended question skipping logic and used this section to share which features they thought were useful or not without regard to their use of the tool (i.e., users who had used the tool shared reasons the tool was not useful). Based on this unexpected pattern of survey responses, we elected to share the absolute numbers of patients who listed each helpful and unhelpful feature and to contextualize these absolute numbers as percentages, with all patients considered in the denominator, with the intent of capturing the helpful and unhelpful features in rank-order.

The ranked order of useful aspects of the educational tool among those who used it were the presence of helpful information (*N* = 184, 72%), ease of navigation (*N* = 156, 61%) and feeling that use of the tool increased the patient’s confidence in the treatment plan (*N* = 100, 39%). Considering both users and non-users of the tool, the ranked order of not useful aspects of the educational tool were patients’ preferences for written material (*N* = 30, 10%), difficulty navigating the application (*N* = 11, 4%), and disliking tablet computers (*N* = 6, 2%).

When free-text survey responses were reviewed, other identified barriers to use were the need to log in with each use or another technical issue preventing access (*N* = 3), difficulties connecting to wireless internet at a hotel (*N* = 2) and redundancy with other materials provided on paper or during the consultation (*N* = 2). Free-text responses (*N* = 2) also revealed evidence that patients shared the educational tool with family members.

### Effectiveness

When asked about the adequacy of the information included in the tool, 87% (*N* = 193) of patients thought the amount of information included in the tool was “about right.” Eleven percent (*N* = 25) thought there was not enough information and only 1.4% (*N* = 3) thought the amount of information included was too much.

Most (59%) patients who had used the tool rated their understanding of breast cancer treatments as 8 or higher (median 8, IQR 5.5 to 10) on a 10-point scale, with 10 indicating the highest level of understanding.

Patients who had used the educational tool as part of their interdisciplinary consultation expressed higher levels of confidence post-intervention (average 9.1, median 10, IQR 9–10) when compared to pre-intervention (average 8.3, median 9.0, IQR 8–10). The average increase in confidence (+ 0.8) was statistically significant (*p* < 0.001).

Among women who used the tool, 92% (*N* = 210) would recommend it to other patients.

### Association between confidence and baseline patient characteristics

We observed an association between lower pre-intervention confidence and the following variables: invasive cancer (vs. ductal carcinoma in situ, mean 8.19 vs. 9.12, *p* = 0.013), stage (stepwise inverse relationship with higher stages corresponding to lower confidence, mean 9.16 for stage 0 to mean 7.44 for stage III, *p* = 0.003) and preferred approach of shared decision making (vs. more passive or dominant role, mean 8.10 vs. 8.83, *p* = 0.008). Interestingly, geographic distance was another significant predictor of lower baseline confidence. Baseline confidence was lowest among patients living between 30 and 80 miles from the clinic (second quartile, mean 7.55) and similar for other quartiles of distance considered (means ranging from 8.39 to 8.79, *p* = 0.004). Neither age nor education was predictive of baseline confidence.

In pre-post analysis, the following factors were associated with greater increase in confidence following consultation and use of the tool: distance from clinic (average increase of 1.81 in patients living between 30 and 80 miles away as compared to other distance quartiles, with average increases ranging from 0.25 to 0.55, *p* = 0.002), and preferred shared decision making approach (average + 1.08 vs. + 0.15 points, *p* = 0.006). Age, education, cancer type and cancer stage were not associated with the magnitude of change in decision confidence following consultation and use of the tool. In a linear regression model predicting change in confidence, the pre-intervention confidence level was the only significant factor (*p* < 0.0001) when adjusted for distance, cancer type, stage, preferred role in decision making, and education. In other words, patients with lower baseline confidence were more likely to experience a positive increase in confidence.

**Fig. 4:**
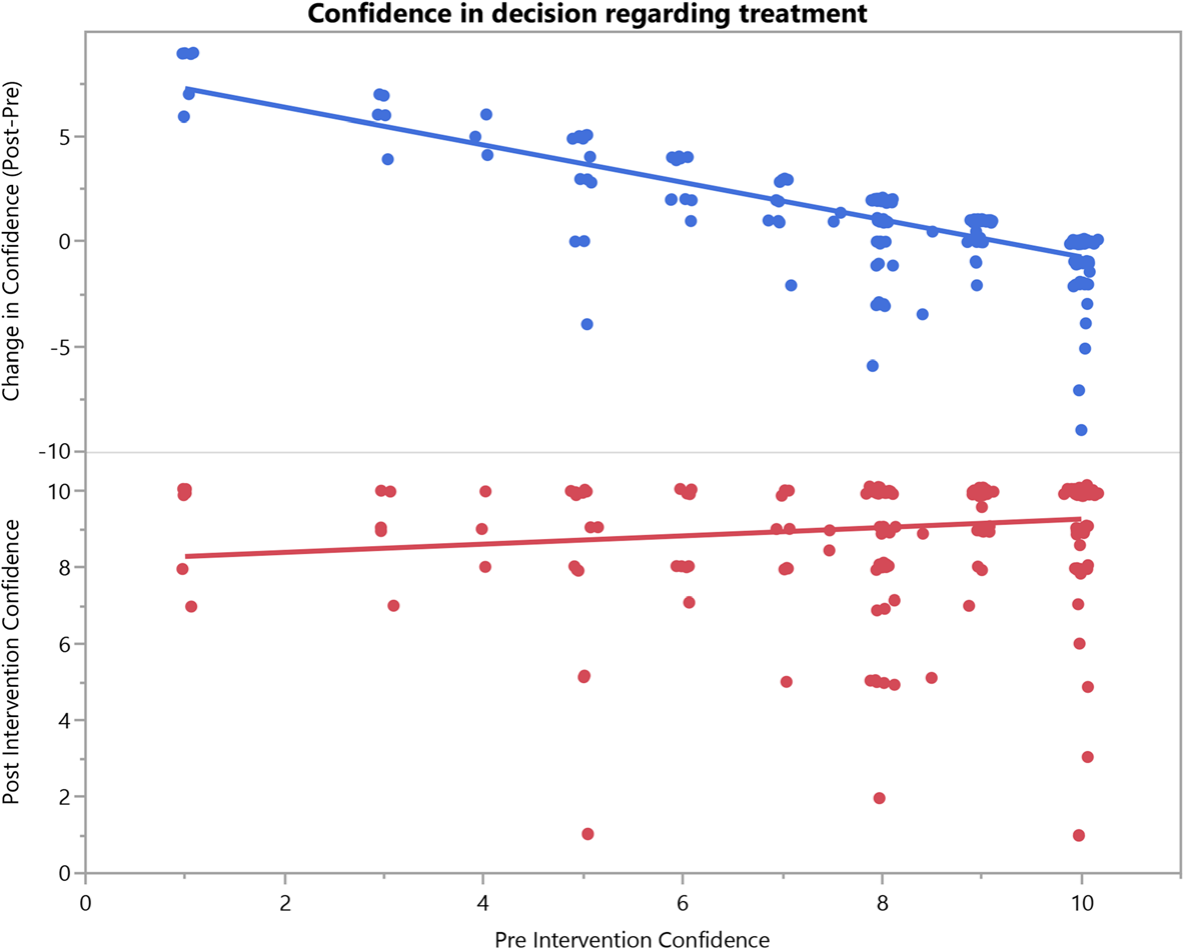
Scatter plot and linear regression for change in confidence and post-intervention confidence against pre-intervention confidence

We therefore concluded that significant associations between baseline characteristics and change in confidence were due to low baseline confidence. Patients with high confidence at baseline were subject to a ceiling effect due to high baseline scores, whereas patients with lower confidence at baseline had a larger range of possible improvements. The graphs in Fig. 4 support this conclusion. A plot of post-treatment confidence against pre-treatment confidence reveals a nearly horizontal line (bottom half of Fig. 4, *r* = + 0.15), suggesting that most patients achieve a high level of confidence regardless of their pre-intervention confidence. Furthermore, when we plotted the change in confidence against pre-intervention confidence, we observed a negative association (top half of Fig. 4, *r* = − 0.77). In other words, patients with lower baseline confidence appeared to have larger gains in confidence.

## Discussion

We have described the implementation of a personalized, electronic patient education and decision making tool in a multidisciplinary breast clinic. The application’s use, in conjunction with clinical consultation, was associated with a significant increase in decision making confidence. In this section, we will discuss the implications of our findings and limitations that apply to our results.

### Primary findings

Mobile applications and decision aids are becoming increasingly popular tools for clinical decision making. We report the successful implementation and integration of a personalized breast cancer educational tool in the form of a mobile tablet application within our breast clinic. We implemented an iOS-based web application in our multidisciplinary Breast Clinic and conducted pre-post surveys to capture patients’ experiences using the application and coupled survey responses with clinical data for analysis. The patient age range was 29 to 85 years. Patients shared the tool with family members and friends who were involved in their care and largely preferred a shared decision making approach. We did not observe differences in treatment options chosen between patients who had and had not used the tool.

We identified features of the tool that were barriers and facilitators of use. Barriers included the need for an active internet connection, the need to log in with a username and password each time the application is accessed, preference for printed materials and difficulties navigating the tool. As our average patient age was 57 years, it is unclear whether these barriers might be most applicable to older patients who, as a whole, may be less accustomed to interaction with digital applications compared to younger patients. Notwithstanding, patients up to 85 years of age used the tool, with 94% saying they would recommend it to other patients like them. Among patients who used the tool, confidence increased by an average of 0.8 points on a 10 point scale (*p* < 0.001).

Facilitators of use included the inclusion of useful educational content, ease of navigation and perceived favorable impact on information comprehension. While adequate information about treatment options is necessary to make a decision, it certainly is not sufficient. Patients must have discussions with their health care providers about personal circumstances, preferences, and values which might make one option more appropriate for them than another. Furthermore, patients may seek to involve family members and friends in the decision making process [11]. With this in mind, the goal of this interactive educational tool is not to replace in-person consultation with a patient’s breast cancer treatment team but rather to enhance patients’ knowledge so that they can be better-prepared for discussions with their providers. To this end, it was designed as a supplemental tool that patients are able to use to review the options available to them whenever, wherever and with whomever they wish. Most (*N* = 276; 76%) women rated their confidence about their final treatment decision between a 9 and 10 on a 10-point scale.

Exploratory analyses revealed that patients with more invasive cancer, higher stage cancer, and patients who preferred a shared decision making decision making approach had lower baseline confidence. It is plausible that patients with invasive or higher stage cancers may experience greater uncertainty about their options. Likewise, patients who prefer shared decision making may feel more uncertain at the time of their initial consultation compared to others because they lack preconceived notions about the existence of a single-best treatment option that they have either already chosen or which they expect their doctor will choose for them.

Although baseline characteristics may predict low baseline confidence, most patients will attain a high level of confidence in their decision following consultation and use of the tool. This suggests that the combination of consultation and the tool may serve as an “equalizer” to increase confidence in patients who are unsure about their decisions, but it has little effect on the confidence of those who already feel confident. A “ceiling effect” or regression toward the mean could alternatively account for this observation.

### Strengths and limitations of the application

Some key components which make the educational tool unique and drive patient satisfaction are the electronic delivery method and personalization of content displayed to the patient based on individual characteristics (Table 2). Although this personalization takes additional effort on the part of the health care provider, the ability to tailor the information reduces information overload and helps the patient focus on only the information that is relevant to her.

**Table 2:** Strengths, Weaknesses, Opportunities Threats (SWOT) analysis

	Helpful	Harmful
**Internal**	**Strengths** • Large sample size • High application uptake • Tablet provided to family (i.e., not required to purchase) • Pre-post responses available • Clinical data available	**Weaknesses** • Limited survey data collected • Lack of pre-implementation period surveys • Pre-post design (vs. randomized) • Attitudes of patients who refused tool not captured • Unable to control for confounders • Limited to iOS-based tablet
**External**	**Opportunities** • SMART on FHIR • Integration into patient record portals • Progressive web application standards • Pre-visit content delivery model • New content formats (e.g., video) • Engaging family members	**Threats** • Generalizability outside of referral population • High baseline confidence • Evolving content delivery models (e.g., standards) and content delivery expectations (e.g., multi-platform)

One important observation was that patients shared the tool with family members and friends who also engaged in the decision making process. The application currently acts as a standalone tool, and patients may choose to share the application with others. A limitation of the educational tool was our inability to integrate it within the electronic health record online portal. In spite of this, we also realized that such integration would limit the ability of others who are involved in decision making to use the application without having access to the patient’s entire medical record through the portal.

Another limitation of the educational tool was that it was formatted only for use on an Apple iPad in landscape orientation. It was not formatted for use on a mobile phone or desktop computer. Non-iOS-based tablets (e.g., Android operating system) were not tested. Additionally, as a web-based application with content stored on a remote server and delivered on-demand, use of the application required an active internet connection. The application may be improved by accommodating additional screen aspect ratios and downloading content onto the tablet so that it may be viewed at times where an active internet connection is not available.

The study involved patients at a highly-specialized referral center. The study results may not generalize to other centers or patient populations. Finally, outcomes were limited to survey responses. We purposefully did not aim to measure impact on treatment decisions made (e.g., affect rates of specific surgical interventions) because the goal of the tool was to facilitate decisions which were concordant with patients’ values and preferences rather than to bias decisions in favor of one option over another.

The current application interface was designed exclusively for our breast cancer patients. However, our success implementing the application within the breast clinic has led to providers in other specialties expressing interest in adapting the application for use with other patient populations. Currently, there is no easy way to port content for other diseases into the application interface. Ongoing efforts are evaluating the utility and feasibility of re-working the application into a modular, cross-platform plug-and-play application that is usable on any device type and into which content for various diseases can be easily entered to develop a new deployment of the application for another disease. We anticipate that this will create a new paradigm for personalized content delivery within our health care enterprise. New open standards, including Health Level 7 SMART on FHIR [12], may allow applications in the future to pull data from the electronic health record rather than relying on manual entry for each patient. Furthermore, data standards may facilitate integration of applications within online patient medical record portals.

### Strengths and limitations of the study

As a pre-post study based on limited survey data, we are limited in the conclusions that can be drawn from our results. We did not conduct baseline clinic-wide surveys prior to implementation of the educational tool to assess patients’ experiences with traditional education methods, and we also did not survey patients who refused the educational tool when it was first offered (though we included in our study patients who were offered and accepted the tool but never used it). Implementation of the application in our clinic as a standard-of-care initiative to improve the quality of care delivered, rather than a randomized study, limited our ability to control for confounding variables. We were unable to separate the impact of the delivery method from the content provided. The surveys also collected a limited amount of information and did not use validated tools (e.g., decisional conflict scale [13]). Our rationale for collecting limited information in surveys for this pilot phase was to ensure that surveys did not impede clinic workflow or impose a significant burden on patients. We also did not collect data on other clinical outcomes because the goal of the tool was to facilitate treatment decisions which were in line with patients’ values and preferences rather than to affect other “hard” clinical outcomes. Baseline confidence was high and a “ceiling effect” may limit our ability to understand the true clinical significance of changes in confidence. It is possible that cognitive biases may influence patients’ perceived confidence levels. For example, patients may feel more confident after being referred to a referral center, and confidence following use of the application could represent confirmation bias, affirming the patient’s decision to be seen at a referral center and to use the application. Additional study is warranted to more fully assess the impact on our clinical practice and patient experience, especially among a more diverse group of patients.

### Accessing health information online

Access to the internet has redefined the way health information is delivered. For example, in a study of female patients with breast cancer, up to 80% used the internet, and 71% searched for information pertaining to their diagnosis [5]. Younger age and higher income are predictors, among others, of a woman’s internet access [14]. However, relatively effortless and quick access to information does not necessarily translate into increased understanding of breast cancer medical, surgical, and radiation therapy options. Internet users may not be proficient in the operational and navigational skills needed to consistently access accurate and trusted health care related information [15]. In addition, as we mentioned earlier, the quality and depth of the presented information may be inadequate, overwhelming or challenging for a layperson to understand. With good reason, about 50% of patients have reservations about the trustworthiness of information obtained on the internet [16]. Although there are a vast number of breast cancer related websites, only a small percentage score “good” in their reliability and in the ability of the information presented to support decision making regarding breast cancer surgery [17, 18]. We feel that mobile interactive and personalized patient educational tools which are endorsed, tailored and provided by the patient’s health care provider represent a preferred approach to electronic content delivery.

### Shared decision making

Many shared decision making experts [11], including colleagues at our institution with whom we have worked, advocate for use of decision aids *during the clinical consultation,* rather than outside of the clinical consultation, to facilitate shared decision making. Our tool does not meet all of the criteria established by the International Patient Decision Aid Standards (IPDAS) Collaboration to be considered a decision aid [19]. We did not set out to develop the tool as a decision aid per se but instead as a patient education tool to enhance patients’ knowledge and help them engage in shared decision making. Shared decision making is paramount for improving outcomes when multiple treatment alternatives are being considered in women with breast cancer [20]. A population-based study of breast cancer survivors found that a patient’s choice of lumpectomy (vs. mastectomy) was strongly associated with higher patient education and the amount of physician-provided information [21]. One key benefit to electronic decision aids is that they allow easy integration of personalized information; however, in spite of this, other considerations may lead to tangible paper-based decision aids being preferred [22].

### Microlearning and videos before the consultation

While our approach incorporated a web-based educational tool that was reviewed *after* the initial consultation, an alternative or complementary approach is provision of education materials *prior* to the initial consultation with the aim of preparing the patient for discussion at the first visit. The feasibility of pre-visit educational videos in patients with breast cancer has been demonstrated elsewhere with favorable patient attitudes [23]. We are currently piloting the approach of a series of concise pre-visit videos in our clinic as an adjunct to the application. Preliminary patient survey responses (unpublished) suggest that this approach is well-received by patients; however, the video creation process may be more costly and cumbersome than generation of written content and may be a limiting factor.

## Conclusions

When used in conjunction with medical consultation, a web-based educational tool for delivery of personalized breast cancer education was associated with an increase in decision making confidence. Most patients thought the application included helpful information, was easy to navigate, and included the right amount of information. Whether distal outcomes are affected is unclear. The personalization afforded by mobile applications may facilitate a new paradigm for clinical decision making tools.

## Data Availability

In order to preserve patient confidentiality, the Institutional Review Board approval for this study does not permit external sharing of the study dataset. Therefore, the datasets generated and/or analyzed during the current study are not publicly available.

## Abbreviations

FHIR: Fast Healthcare Interoperability Resources
IPDAS: International Patient Decision Aid Standards
IQR: Interquartile range
r: Pearson correlation coefficient
SD: Standard deviation
SMART: Substitutable Medical Apps, Reusable Technology
